# 2-(1-Amino-4-*tert*-butyl­cyclo­hex­yl)acetic acid (tBu-β^3,3^-Ac_6_c) hemihydrate^1^

**DOI:** 10.1107/S1600536813012725

**Published:** 2013-05-15

**Authors:** Naiem Ahmad Wani, Vivek K. Gupta, Rajni Kant, Subrayashastry Aravinda, Rajkishor Rai

**Affiliations:** a Medicinal Chemistry Division, Indian Institute of Integrative Medicine, Canal Road, Jammu Tawi 180 001, India; b X-ray Crystallography Laboratory, Post-Graduate Department of Physics & Electronics, University of Jammu, Jammu Tawi 180 006, India

## Abstract

The title compound, C_12_H_23_NO_2_·0.5H_2_O, crystallized with two 2-(1-amino-4-tert-butylcyclohexyl)acetic acid mol­ecules, which are present as zwitterions, and one water mol­ecule in the asymmetric unit. The mol­ecular structure of each zwitterion is stabilized by an intra­molecular six-membered (*C*_6_ ) N—H⋯O hydrogen bond. In the crystal, the two independent zwitterions are linked head-to-head by N—H⋯O hydrogen bonds. Further O—H⋯O and N—H⋯O hydrogen bonds link the zwitterions and the water molecules, forming sandwich-like layers, with a hydrophilic filling and a hydrophobic exterior, lying parallel to the *ab* plane.

## Related literature

For the importance of β-amino acids, see: Politi *et al.* (2009 ▶); Jiang & Yu (2007 ▶); Hansen *et al.* (2011 ▶). For related structures, see: Seebach *et al.* (1998 ▶); Vasudev *et al.* (2008 ▶, 2009 ▶). 
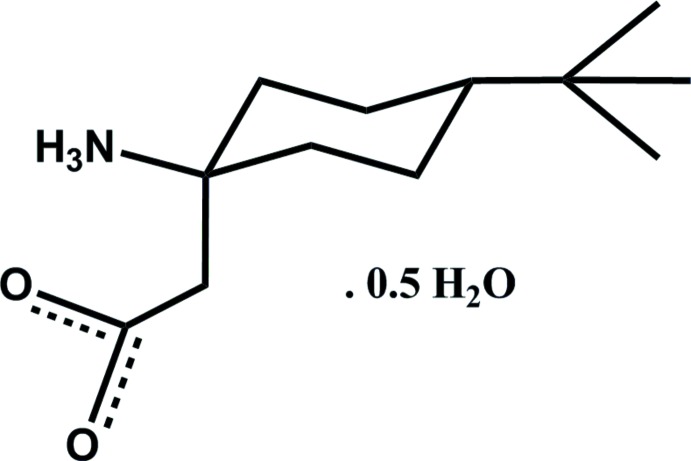


## Experimental

### 

#### Crystal data

C_12_H_23_NO_2_·0.5H_2_O
*M* *_r_* = 222.32
Triclinic, 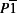

*a* = 6.4164 (2) Å
*b* = 10.8091 (3) Å
*c* = 19.1335 (6) Å
α = 96.843 (3)°
β = 92.018 (3)°
γ = 93.901 (3)°
*V* = 1313.25 (7) Å^3^
*Z* = 4
Mo *K*α radiation
μ = 0.08 mm^−1^
*T* = 293 K
0.3 × 0.08 × 0.08 mm

#### Data collection

Oxford Diffraction Xcalibur Sapphire3 diffractometer
Absorption correction: multi-scan (*CrysAlis PRO*; Oxford Diffraction, 2010 ▶) *T*_min_ = 0.830, *T*_max_ = 1.000
22269 measured reflections
5701 independent reflections
3628 reflections with *I* > 2σ(*I*)
*R*_int_ = 0.048

#### Refinement

*R*[*F* ^2^ > 2σ(*F* ^2^)] = 0.053
*wR*(*F* ^2^) = 0.141
*S* = 1.02
5701 reflections
472 parameters
All H-atom parameters refined
Δρ_max_ = 0.21 e Å^−3^
Δρ_min_ = −0.17 e Å^−3^

### 

Data collection: *CrysAlis PRO* (Oxford Diffraction, 2010 ▶); cell refinement: *CrysAlis PRO*; data reduction: *CrysAlis PRO*; program(s) used to solve structure: *SHELXS97* (Sheldrick, 2008 ▶); program(s) used to refine structure: *SHELXL97* (Sheldrick, 2008 ▶); molecular graphics: *ORTEP-3 for Windows* (Farrugia, 2012 ▶); software used to prepare material for publication: *PLATON* (Spek, 2009 ▶).

## Supplementary Material

Crystal structure: contains datablock(s) I, global. DOI: 10.1107/S1600536813012725/qm2098sup1.cif

Structure factors: contains datablock(s) I. DOI: 10.1107/S1600536813012725/qm2098Isup2.hkl

Supplementary material file. DOI: 10.1107/S1600536813012725/qm2098Isup3.cml

Additional supplementary materials:  crystallographic information; 3D view; checkCIF report

## Figures and Tables

**Table 1:** Hydrogen-bond geometry (Å, °)

*D*—H⋯*A*	*D*—H	H⋯*A*	*D*⋯*A*	*D*—H⋯*A*
N1—H1*N*⋯O2	1.00 (2)	2.12 (2)	2.792 (2)	123.5 (15)
N1—H1*N*⋯O1*W* ^i^	1.00 (2)	2.11 (2)	2.919 (2)	137.8 (17)
O1*W*—H1*W*⋯O1	0.89 (3)	2.03 (3)	2.903 (2)	166 (3)
N1—H2*N*⋯O4^ii^	0.96 (2)	1.81 (2)	2.747 (2)	166.0 (17)
O1*W*—H2*W*⋯O3^iii^	0.90 (4)	2.04 (4)	2.929 (2)	169 (3)
N1—H3*N*⋯O3	0.97 (2)	1.86 (2)	2.7903 (19)	160.7 (17)
N2—H4*N*⋯O2^i^	1.00 (2)	1.73 (2)	2.729 (2)	170.5 (19)
N2—H5*N*⋯O1^iii^	0.99 (2)	1.818 (19)	2.779 (2)	163.1 (18)
N2—H6*N*⋯O3	0.93 (2)	2.10 (2)	2.836 (2)	135.4 (17)

Symmetry codes: (i) 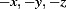; (ii) 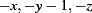; (iii) 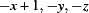.

**Fig. 1.**
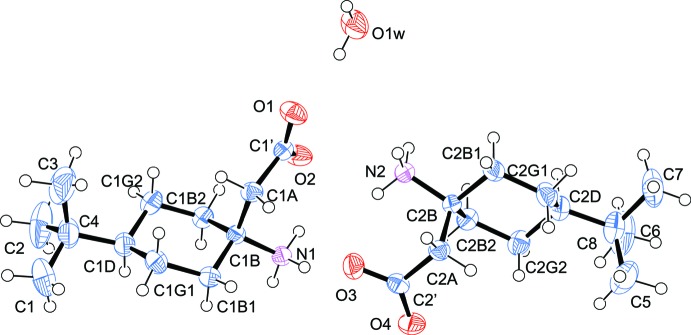
The molecular structure of the compound. The thermal ellipsoids are drawn at the 40% probability level. H atoms are shown as small spheres of arbitrary radii.

**Fig. 2.**
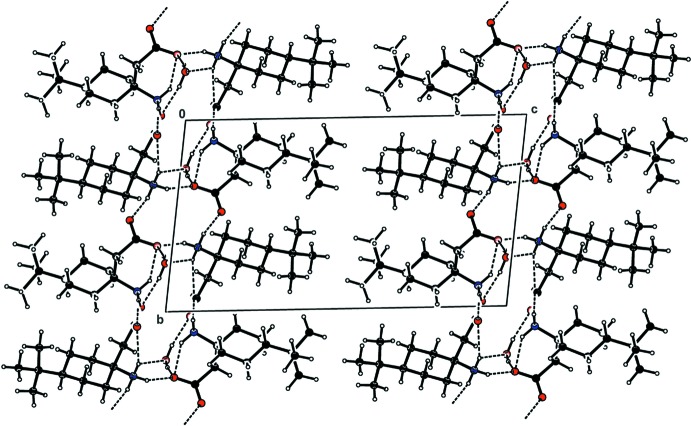
The packing arrangement of molecules viewed down the a-axis. Hydrogen bonds are shown in dotted lines.

**Fig. 3.**
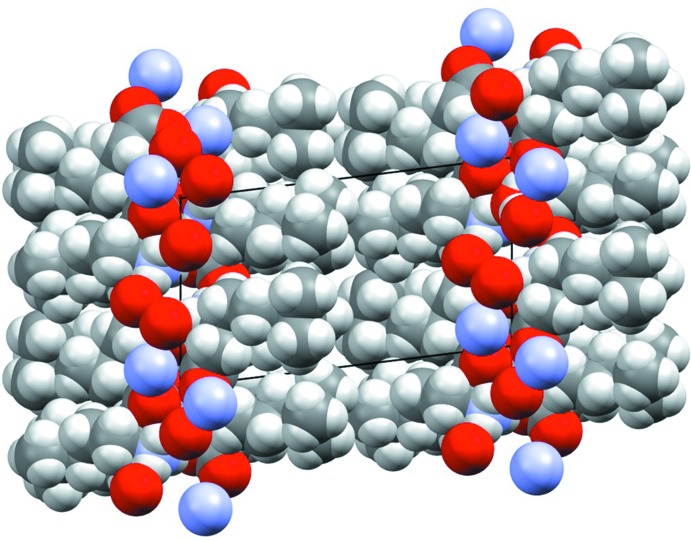
The space filling model showing the alternative hydrophobic and hydrophilic layers in crystal lattice.

## supplementary crystallographic information

### Comment

Disubstituted β-amino acids have been used as building blocks in potent pharmaceutical drugs and functional materials (Politi *et al.*, 2009; Jiang & Yu, 2007). The use of disubstituted β-amino acids has been reported to give highly potent antimicrobial β-peptidomimetics with exceptional proteolytic stability and low hemolytic activity (Hansen *et al.*, 2011). Geminally disubstituted β-amino acids have been synthesized and characterized (Seebach *et al.* 1998; Vasudev *et al.*, 2008). The present report describes the molecular structure of 2-(1-amino-4-*tert*-butylcyclohexyl) acetic acid (tBu-β^3,3^-Ac_6_c) as shown in Fig. 1. tBu-β^3,3^-Ac_6_c is considered to be a homologue of 4-tertiarybutylgabapentin (Vasudev *et al.*, 2009). The compound crystallized in space group *P*1. The molecular conformation of tBu-β^3,3^-Ac_6_c is shown in Fig. 2. The crystal structure shows a six membered (C_6_) NH···O intramolecular hydrogen bond between NH and the carbonyl group of tBu-β^3,3^-Ac_6_c. In the structure the cyclohexane ring adopts a chair conformation with equatorial *tert*-butyl and amino groups. The carboxymethyl group occupies the axial position. Fig. 3 shows the packing of molecules in the crystal. Intermolecular O···HO and NH···O hydrogen bonds stabilize the structure leading to the formation of hydrophobic and hydrophilic layers as shown in Fig. 3.

### Experimental

To a solution of 4-*tert*-cyclohexanone (7.70 g, 50 mmol), malonic acid (5.20 g, 50 mmol) in 100 ml of ethanol was added 11.55 g (150 mmol) of ammonium acetate. The reaction mixture was refluxed for 24 h. After completion of the reaction, the reaction mixture was allowed to cool to room temperature and ethyl alcohol was evaporated under vacuum. The residue was triturated with acetone (3 *x* 50 ml) and dried to yield a white solid (Yield 6.5 g, 61%). M.P. 265–267°C. Single crystals suitable for X-ray diffraction were obtained by slow evaporation of a methanol/water (8:2) mixture.

### Refinement

H atoms were located in a difference Fourier map and both their coordinrates and *U*_iso_ were refined.

### Crystal data

**Table:**

C_12_H_23_NO_2_·0.5H_2_O	*Z* = 4
*M**_r_* = 222.32	*F*(000) = 492
Triclinic, *P*1	*D*_x_ = 1.124 Mg m^−^^3^
Hall symbol: -P 1	Mo *K*α radiation, λ = 0.71073 Å
*a* = 6.4164 (2) Å	Cell parameters from 8184 reflections
*b* = 10.8091 (3) Å	θ = 3.4–27.0°
*c* = 19.1335 (6) Å	µ = 0.08 mm^−^^1^
α = 96.843 (3)°	*T* = 293 K
β = 92.018 (3)°	Needle, color less
γ = 93.901 (3)°	0.3 × 0.08 × 0.08 mm
*V* = 1313.25 (7) Å^3^	

### Data collection

**Table:**

Oxford Diffraction Xcalibur Sapphire3 diffractometer	5701 independent reflections
Radiation source: fine-focus sealed tube	3628 reflections with *I* > 2σ(*I*)
Graphite monochromator	*R*_int_ = 0.048
Detector resolution: 16.1049 pixels mm^-1^	θ_max_ = 27.0°, θ_min_ = 3.4°
ω scan	*h* = −8→8
Absorption correction: multi-scan (*CrysAlis PRO*; Oxford Diffraction, 2010)	*k* = −13→13
*T*_min_ = 0.830, *T*_max_ = 1.000	*l* = −24→24
22269 measured reflections	

### Refinement

**Table:**

Refinement on *F*^2^	Primary atom site location: structure-invariant direct methods
Least-squares matrix: full	Secondary atom site location: difference Fourier map
*R*[*F*^2^ > 2σ(*F*^2^)] = 0.053	Hydrogen site location: inferred from neighbouring sites
*wR*(*F*^2^) = 0.141	All H-atom parameters refined
*S* = 1.02	*w* = 1/[σ^2^(*F*_o_^2^) + (0.0691*P*)^2^] where *P* = (*F*_o_^2^ + 2*F*_c_^2^)/3
5701 reflections	(Δ/σ)_max_ < 0.001
472 parameters	Δρ_max_ = 0.21 e Å^−^^3^
0 restraints	Δρ_min_ = −0.17 e Å^−^^3^

### Special details

**Table:**

Experimental. *CrysAlis PRO*, Oxford Diffraction Ltd., Version 1.171.34.40 (release 27–08-2010 CrysAlis171. NET) (compiled Aug 27 2010,11:50:40) Empirical absorption correction using spherical harmonics, implemented in SCALE3 ABSPACK scaling algorithm.
Geometry. All e.s.d.'s (except the e.s.d. in the dihedral angle between two l.s. planes) are estimated using the full covariance matrix. The cell e.s.d.'s are taken into account individually in the estimation of e.s.d.'s in distances, angles and torsion angles; correlations between e.s.d.'s in cell parameters are only used when they are defined by crystal symmetry. An approximate (isotropic) treatment of cell e.s.d.'s is used for estimating e.s.d.'s involving l.s. planes.
Refinement. Refinement of *F*^2^ against ALL reflections. The weighted *R*-factor *wR* and goodness of fit *S* are based on *F*^2^, conventional *R*-factors *R* are based on *F*, with *F* set to zero for negative *F*^2^. The threshold expression of *F*^2^ > σ(*F*^2^) is used only for calculating *R*-factors(gt) *etc*. and is not relevant to the choice of reflections for refinement. *R*-factors based on *F*^2^ are statistically about twice as large as those based on *F*, and *R*- factors based on ALL data will be even larger.

### Fractional atomic coordinates and isotropic or equivalent isotropic displacement parameters (Å^2^)

**Table:**

	*x*	*y*	*z*	*U*_iso_*/*U*_eq_	
H7B2	−0.105 (2)	−0.1438 (16)	−0.1669 (8)	0.024 (4)*	
H2N	−0.023 (3)	−0.403 (2)	0.0878 (10)	0.054 (6)*	
H1D	0.244 (3)	−0.3888 (17)	0.2864 (9)	0.037 (5)*	
H8B2	−0.085 (2)	−0.2797 (16)	−0.1450 (9)	0.028 (4)*	
H5B1	0.436 (3)	−0.0776 (16)	−0.2082 (8)	0.031 (4)*	
H2B1	0.250 (3)	−0.4631 (17)	0.1574 (9)	0.033 (5)*	
H4B2	−0.002 (3)	−0.1735 (18)	0.1900 (9)	0.041 (5)*	
H3B2	−0.022 (3)	−0.3159 (17)	0.2047 (8)	0.030 (4)*	
H1B1	0.434 (3)	−0.4057 (17)	0.1126 (10)	0.044 (5)*	
H2A1	0.448 (3)	−0.2078 (18)	0.0782 (11)	0.052 (6)*	
H4A2	0.329 (3)	−0.3383 (17)	−0.2085 (10)	0.036 (5)*	
H6G1	0.313 (3)	−0.2261 (17)	−0.3029 (9)	0.035 (5)*	
H2D	−0.074 (3)	−0.0963 (19)	−0.2916 (10)	0.049 (5)*	
H3N	0.143 (3)	−0.3572 (18)	0.0340 (11)	0.048 (6)*	
H4N	0.117 (3)	−0.047 (2)	−0.0832 (10)	0.057 (6)*	
H1G1	0.587 (3)	−0.281 (2)	0.2142 (10)	0.053 (6)*	
H6B1	0.213 (3)	−0.0148 (19)	−0.2038 (10)	0.051 (6)*	
H1A1	0.445 (3)	−0.1392 (18)	0.1528 (10)	0.050 (5)*	
H3G2	0.313 (3)	−0.1358 (19)	0.2638 (10)	0.048 (5)*	
H1N	−0.034 (3)	−0.271 (2)	0.0632 (12)	0.072 (7)*	
H3A1	0.488 (3)	−0.2635 (18)	−0.1485 (10)	0.049 (5)*	
H6N	0.209 (3)	−0.164 (2)	−0.0561 (11)	0.055 (6)*	
H7G2	−0.234 (3)	−0.2967 (19)	−0.2592 (10)	0.054 (6)*	
H8G2	−0.005 (3)	−0.3538 (19)	−0.2648 (10)	0.051 (6)*	
H2G1	0.557 (3)	−0.422 (2)	0.2258 (10)	0.053 (6)*	
H5G1	0.279 (3)	−0.0874 (19)	−0.3186 (11)	0.051 (6)*	
H5N	0.366 (3)	−0.0632 (19)	−0.0826 (10)	0.053 (6)*	
H4G2	0.125 (3)	−0.187 (2)	0.3059 (12)	0.059 (6)*	
H1W	0.271 (5)	0.177 (3)	0.0290 (17)	0.111 (11)*	
H2W	0.394 (6)	0.291 (3)	0.0225 (17)	0.123 (12)*	
H11	−0.018 (4)	−0.413 (3)	−0.3834 (14)	0.085 (9)*	
H10	−0.021 (4)	−0.368 (3)	−0.4636 (17)	0.104 (9)*	
H4	0.400 (5)	−0.273 (3)	0.4587 (19)	0.112 (10)*	
H8	0.709 (5)	−0.173 (3)	0.3235 (18)	0.108 (12)*	
H9	0.702 (4)	−0.168 (3)	0.4047 (16)	0.094 (8)*	
H17	−0.048 (4)	−0.147 (3)	−0.4749 (16)	0.097 (9)*	
H13	−0.349 (4)	−0.318 (3)	−0.3683 (16)	0.098 (10)*	
H7	0.537 (6)	−0.094 (4)	0.359 (2)	0.144 (15)*	
H18	0.173 (5)	−0.118 (3)	−0.4312 (16)	0.118 (11)*	
H15	−0.348 (5)	−0.260 (3)	−0.443 (2)	0.122 (11)*	
H3	0.657 (5)	−0.401 (3)	0.4112 (18)	0.111 (10)*	
H1	0.719 (8)	−0.425 (5)	0.328 (3)	0.21 (2)*	
H12	0.184 (5)	−0.338 (3)	−0.4072 (16)	0.117 (12)*	
H2	0.511 (6)	−0.477 (4)	0.358 (2)	0.145 (17)*	
H16	−0.049 (5)	−0.039 (3)	−0.4076 (16)	0.113 (11)*	
H14	−0.376 (6)	−0.163 (4)	−0.379 (2)	0.157 (17)*	
H5	0.257 (7)	−0.196 (4)	0.412 (2)	0.17 (2)*	
H6	0.221 (7)	−0.341 (4)	0.408 (2)	0.152 (19)*	
N1	0.0633 (2)	−0.33395 (15)	0.07518 (8)	0.0329 (4)	
C1B	0.2122 (2)	−0.28252 (15)	0.13598 (8)	0.0287 (4)	
C1A	0.3477 (3)	−0.17329 (16)	0.11343 (10)	0.0324 (4)	
C1'	0.2390 (3)	−0.06639 (16)	0.08696 (8)	0.0322 (4)	
O1	0.35298 (19)	0.02596 (12)	0.07388 (7)	0.0465 (4)	
O2	0.04386 (18)	−0.07591 (12)	0.07914 (7)	0.0472 (4)	
C1B1	0.3463 (3)	−0.38803 (18)	0.15219 (10)	0.0363 (4)	
C1G1	0.4799 (3)	−0.3539 (2)	0.22047 (10)	0.0417 (5)	
C1D	0.3481 (3)	−0.31899 (19)	0.28349 (9)	0.0410 (5)	
C1B2	0.0815 (3)	−0.24555 (19)	0.19973 (9)	0.0355 (4)	
C1G2	0.2166 (3)	−0.2123 (2)	0.26764 (10)	0.0417 (5)	
C1	0.6058 (8)	−0.4031 (4)	0.3647 (2)	0.1059 (13)	
C2	0.3211 (7)	−0.2808 (6)	0.41463 (15)	0.1039 (13)	
C3	0.6186 (6)	−0.1766 (4)	0.36012 (18)	0.0875 (10)	
C4	0.4739 (3)	−0.2945 (2)	0.35522 (10)	0.0556 (6)	
N2	0.2266 (3)	−0.10896 (15)	−0.08954 (8)	0.0334 (4)	
C2B	0.2019 (3)	−0.18188 (15)	−0.16194 (8)	0.0295 (4)	
C2A	0.3351 (3)	−0.29428 (16)	−0.16156 (9)	0.0317 (4)	
O3	0.2980 (2)	−0.34430 (12)	−0.04517 (6)	0.0453 (4)	
O4	0.1620 (2)	−0.48186 (12)	−0.13336 (7)	0.0512 (4)	
C2'	0.2591 (3)	−0.38243 (16)	−0.10927 (9)	0.0325 (4)	
C2B1	0.2811 (3)	−0.09492 (18)	−0.21438 (9)	0.0373 (4)	
C2G1	0.2287 (3)	−0.1492 (2)	−0.29083 (10)	0.0411 (5)	
C2D	−0.0054 (3)	−0.18123 (19)	−0.30538 (9)	0.0402 (5)	
C2G2	−0.0794 (3)	−0.2736 (2)	−0.25466 (9)	0.0405 (5)	
C2B2	−0.0309 (3)	−0.21945 (19)	−0.17772 (9)	0.0352 (4)	
C5	0.0283 (6)	−0.3446 (3)	−0.41124 (15)	0.0771 (8)	
C6	−0.3038 (5)	−0.2413 (5)	−0.39376 (16)	0.0922 (12)	
C7	0.0084 (6)	−0.1213 (4)	−0.42883 (15)	0.0823 (9)	
C8	−0.0661 (3)	−0.2238 (2)	−0.38415 (10)	0.0533 (6)	
O1W	0.2635 (3)	0.25362 (19)	0.01566 (8)	0.0595 (4)	

### Atomic displacement parameters (Å^2^)

**Table:**

	*U*^11^	*U*^22^	*U*^33^	*U*^12^	*U*^13^	*U*^23^
N1	0.0354 (8)	0.0291 (9)	0.0332 (8)	−0.0036 (7)	−0.0030 (7)	0.0048 (6)
C1B	0.0292 (8)	0.0266 (9)	0.0296 (9)	0.0006 (7)	−0.0028 (7)	0.0026 (7)
C1A	0.0287 (9)	0.0290 (10)	0.0385 (10)	−0.0006 (8)	−0.0020 (8)	0.0027 (7)
C1'	0.0357 (10)	0.0291 (10)	0.0306 (9)	0.0013 (8)	−0.0030 (7)	0.0005 (7)
O1	0.0416 (7)	0.0380 (8)	0.0615 (9)	−0.0044 (6)	−0.0016 (6)	0.0181 (6)
O2	0.0335 (7)	0.0330 (8)	0.0750 (10)	0.0027 (6)	−0.0116 (6)	0.0102 (6)
C1B1	0.0413 (10)	0.0315 (11)	0.0366 (10)	0.0075 (9)	0.0005 (8)	0.0033 (8)
C1G1	0.0412 (11)	0.0421 (12)	0.0431 (11)	0.0108 (10)	−0.0048 (8)	0.0081 (9)
C1D	0.0435 (11)	0.0432 (12)	0.0355 (10)	−0.0062 (10)	−0.0031 (8)	0.0085 (8)
C1B2	0.0318 (9)	0.0381 (11)	0.0363 (10)	0.0037 (9)	0.0007 (7)	0.0025 (8)
C1G2	0.0415 (11)	0.0471 (13)	0.0344 (10)	0.0043 (10)	0.0020 (8)	−0.0044 (8)
C1	0.140 (3)	0.105 (3)	0.076 (2)	0.028 (3)	−0.051 (2)	0.027 (2)
C2	0.106 (3)	0.169 (4)	0.0339 (15)	−0.008 (3)	−0.0028 (15)	0.0161 (19)
C3	0.086 (2)	0.102 (3)	0.0660 (19)	−0.032 (2)	−0.0347 (17)	0.0090 (17)
C4	0.0604 (13)	0.0670 (15)	0.0389 (11)	−0.0024 (12)	−0.0102 (10)	0.0126 (10)
N2	0.0395 (9)	0.0294 (9)	0.0308 (8)	0.0045 (8)	−0.0030 (6)	0.0022 (6)
C2B	0.0356 (9)	0.0257 (9)	0.0266 (8)	−0.0006 (7)	0.0006 (7)	0.0025 (7)
C2A	0.0346 (10)	0.0288 (10)	0.0320 (10)	0.0012 (8)	0.0059 (7)	0.0037 (7)
O3	0.0559 (8)	0.0475 (8)	0.0324 (7)	−0.0048 (7)	0.0018 (6)	0.0090 (6)
O4	0.0638 (9)	0.0327 (8)	0.0546 (8)	−0.0154 (7)	0.0026 (7)	0.0056 (6)
C2'	0.0303 (9)	0.0301 (10)	0.0388 (10)	0.0049 (8)	0.0042 (7)	0.0083 (8)
C2B1	0.0415 (11)	0.0330 (11)	0.0373 (10)	−0.0047 (9)	−0.0010 (8)	0.0097 (8)
C2G1	0.0448 (11)	0.0447 (12)	0.0347 (10)	−0.0058 (10)	0.0024 (8)	0.0134 (9)
C2D	0.0419 (11)	0.0464 (12)	0.0324 (10)	0.0047 (9)	−0.0008 (8)	0.0042 (8)
C2G2	0.0345 (10)	0.0478 (13)	0.0369 (10)	−0.0060 (10)	−0.0005 (8)	0.0009 (8)
C2B2	0.0329 (9)	0.0375 (11)	0.0350 (10)	0.0012 (9)	0.0038 (7)	0.0031 (8)
C5	0.094 (2)	0.083 (2)	0.0490 (16)	0.0087 (18)	−0.0020 (15)	−0.0152 (14)
C6	0.0590 (17)	0.163 (4)	0.0507 (17)	−0.003 (2)	−0.0195 (13)	0.011 (2)
C7	0.105 (3)	0.107 (3)	0.0371 (14)	0.008 (2)	−0.0058 (14)	0.0227 (15)
C8	0.0503 (12)	0.0753 (16)	0.0332 (10)	0.0041 (11)	−0.0039 (9)	0.0040 (10)
O1W	0.0497 (10)	0.0632 (11)	0.0667 (10)	−0.0001 (8)	−0.0156 (7)	0.0203 (8)

### Geometric parameters (Å, º)

**Table:**

N1—C1B	1.507 (2)	N2—H4N	1.00 (2)
N1—H2N	0.96 (2)	N2—H6N	0.93 (2)
N1—H3N	0.97 (2)	N2—H5N	0.99 (2)
N1—H1N	0.99 (2)	C2B—C2B1	1.531 (2)
C1B—C1B1	1.529 (2)	C2B—C2B2	1.531 (2)
C1B—C1A	1.531 (2)	C2B—C2A	1.532 (2)
C1B—C1B2	1.534 (2)	C2A—C2'	1.531 (2)
C1A—C1'	1.517 (2)	C2A—H4A2	0.962 (18)
C1A—H2A1	1.01 (2)	C2A—H3A1	1.03 (2)
C1A—H1A1	0.98 (2)	O3—C2'	1.257 (2)
C1'—O1	1.252 (2)	O4—C2'	1.236 (2)
C1'—O2	1.252 (2)	C2B1—C2G1	1.527 (3)
C1B1—C1G1	1.530 (3)	C2B1—H5B1	0.997 (17)
C1B1—H2B1	1.004 (18)	C2B1—H6B1	1.00 (2)
C1B1—H1B1	0.968 (19)	C2G1—C2D	1.526 (3)
C1G1—C1D	1.522 (3)	C2G1—H6G1	1.032 (18)
C1G1—H1G1	1.03 (2)	C2G1—H5G1	0.95 (2)
C1G1—H2G1	0.93 (2)	C2D—C2G2	1.537 (3)
C1D—C1G2	1.527 (3)	C2D—C8	1.551 (3)
C1D—C4	1.553 (3)	C2D—H2D	1.06 (2)
C1D—H1D	0.981 (18)	C2G2—C2B2	1.531 (2)
C1B2—C1G2	1.528 (3)	C2G2—H7G2	1.00 (2)
C1B2—H4B2	1.01 (2)	C2G2—H8G2	1.02 (2)
C1B2—H3B2	0.992 (17)	C2B2—H7B2	0.977 (17)
C1G2—H3G2	1.01 (2)	C2B2—H8B2	1.009 (17)
C1G2—H4G2	0.98 (2)	C5—C8	1.520 (4)
C1—C4	1.518 (4)	C5—H11	1.00 (3)
C1—H3	0.94 (3)	C5—H10	1.04 (3)
C1—H1	1.04 (5)	C5—H12	1.00 (3)
C1—H2	0.96 (4)	C6—C8	1.526 (3)
C2—C4	1.527 (4)	C6—H13	1.04 (3)
C2—H4	0.96 (4)	C6—H15	0.96 (4)
C2—H5	1.04 (5)	C6—H14	1.01 (4)
C2—H6	0.88 (4)	C7—C8	1.539 (4)
C3—C4	1.515 (4)	C7—H17	0.94 (3)
C3—H8	0.93 (3)	C7—H18	1.06 (3)
C3—H9	0.98 (3)	C7—H16	1.03 (3)
C3—H7	1.06 (4)	O1W—H1W	0.90 (3)
N2—C2B	1.508 (2)	O1W—H2W	0.90 (4)
			
C1B—N1—H2N	109.8 (12)	C2B—N2—H6N	108.7 (13)
C1B—N1—H3N	109.1 (11)	H4N—N2—H6N	108.0 (17)
H2N—N1—H3N	112.0 (16)	C2B—N2—H5N	110.6 (11)
C1B—N1—H1N	111.5 (13)	H4N—N2—H5N	108.7 (16)
H2N—N1—H1N	106.4 (17)	H6N—N2—H5N	110.4 (17)
H3N—N1—H1N	108.0 (17)	N2—C2B—C2B1	107.29 (13)
N1—C1B—C1B1	107.24 (14)	N2—C2B—C2B2	108.19 (14)
N1—C1B—C1A	108.17 (13)	C2B1—C2B—C2B2	109.69 (14)
C1B1—C1B—C1A	110.82 (14)	N2—C2B—C2A	107.30 (14)
N1—C1B—C1B2	107.71 (14)	C2B1—C2B—C2A	111.09 (14)
C1B1—C1B—C1B2	109.03 (14)	C2B2—C2B—C2A	113.05 (14)
C1A—C1B—C1B2	113.62 (14)	C2'—C2A—C2B	112.01 (13)
C1'—C1A—C1B	118.24 (14)	C2'—C2A—H4A2	110.0 (10)
C1'—C1A—H2A1	110.8 (11)	C2B—C2A—H4A2	108.2 (10)
C1B—C1A—H2A1	108.4 (11)	C2'—C2A—H3A1	109.0 (11)
C1'—C1A—H1A1	108.7 (11)	C2B—C2A—H3A1	109.5 (11)
C1B—C1A—H1A1	108.7 (11)	H4A2—C2A—H3A1	108.1 (14)
H2A1—C1A—H1A1	100.6 (15)	O4—C2'—O3	126.20 (16)
O1—C1'—O2	124.32 (16)	O4—C2'—C2A	117.73 (16)
O1—C1'—C1A	116.98 (15)	O3—C2'—C2A	116.04 (15)
O2—C1'—C1A	118.70 (15)	C2G1—C2B1—C2B	112.51 (15)
C1B—C1B1—C1G1	112.15 (15)	C2G1—C2B1—H5B1	108.3 (9)
C1B—C1B1—H2B1	107.8 (10)	C2B—C2B1—H5B1	110.5 (10)
C1G1—C1B1—H2B1	108.6 (10)	C2G1—C2B1—H6B1	109.5 (11)
C1B—C1B1—H1B1	107.4 (11)	C2B—C2B1—H6B1	107.1 (11)
C1G1—C1B1—H1B1	110.6 (11)	H5B1—C2B1—H6B1	108.9 (15)
H2B1—C1B1—H1B1	110.2 (15)	C2B1—C2G1—C2D	112.37 (16)
C1D—C1G1—C1B1	112.19 (16)	C2B1—C2G1—H6G1	108.9 (10)
C1D—C1G1—H1G1	110.3 (11)	C2D—C2G1—H6G1	111.6 (10)
C1B1—C1G1—H1G1	109.0 (11)	C2B1—C2G1—H5G1	105.8 (12)
C1D—C1G1—H2G1	112.6 (12)	C2D—C2G1—H5G1	111.0 (12)
C1B1—C1G1—H2G1	106.3 (13)	H6G1—C2G1—H5G1	106.8 (15)
H1G1—C1G1—H2G1	106.2 (16)	C2G1—C2D—C2G2	108.11 (15)
C1G1—C1D—C1G2	108.70 (16)	C2G1—C2D—C8	113.58 (15)
C1G1—C1D—C4	114.32 (17)	C2G2—C2D—C8	114.75 (16)
C1G2—C1D—C4	114.12 (17)	C2G1—C2D—H2D	104.7 (10)
C1G1—C1D—H1D	107.8 (10)	C2G2—C2D—H2D	108.2 (10)
C1G2—C1D—H1D	103.7 (10)	C8—C2D—H2D	106.9 (10)
C4—C1D—H1D	107.4 (10)	C2B2—C2G2—C2D	111.36 (16)
C1G2—C1B2—C1B	112.20 (15)	C2B2—C2G2—H7G2	106.5 (11)
C1G2—C1B2—H4B2	110.7 (10)	C2D—C2G2—H7G2	113.4 (12)
C1B—C1B2—H4B2	109.2 (10)	C2B2—C2G2—H8G2	108.8 (11)
C1G2—C1B2—H3B2	110.2 (10)	C2D—C2G2—H8G2	109.1 (11)
C1B—C1B2—H3B2	108.5 (9)	H7G2—C2G2—H8G2	107.6 (16)
H4B2—C1B2—H3B2	105.8 (14)	C2B—C2B2—C2G2	112.75 (14)
C1D—C1G2—C1B2	112.06 (16)	C2B—C2B2—H7B2	106.6 (9)
C1D—C1G2—H3G2	109.0 (11)	C2G2—C2B2—H7B2	109.6 (9)
C1B2—C1G2—H3G2	109.7 (11)	C2B—C2B2—H8B2	111.1 (9)
C1D—C1G2—H4G2	112.5 (12)	C2G2—C2B2—H8B2	110.5 (9)
C1B2—C1G2—H4G2	108.1 (12)	H7B2—C2B2—H8B2	106.0 (13)
H3G2—C1G2—H4G2	105.2 (16)	C8—C5—H11	110.8 (15)
C4—C1—H3	112 (2)	C8—C5—H10	107.8 (16)
C4—C1—H1	119 (3)	H11—C5—H10	110 (2)
H3—C1—H1	112 (3)	C8—C5—H12	112.9 (19)
C4—C1—H2	106 (2)	H11—C5—H12	106 (2)
H3—C1—H2	103 (3)	H10—C5—H12	110 (2)
H1—C1—H2	103 (4)	C8—C6—H13	106.1 (16)
C4—C2—H4	108.2 (19)	C8—C6—H15	111 (2)
C4—C2—H5	106 (2)	H13—C6—H15	108 (3)
H4—C2—H5	106 (3)	C8—C6—H14	113 (2)
C4—C2—H6	111 (3)	H13—C6—H14	115 (3)
H4—C2—H6	116 (3)	H15—C6—H14	103 (3)
H5—C2—H6	109 (4)	C8—C7—H17	106.2 (17)
C4—C3—H8	115 (2)	C8—C7—H18	109.3 (18)
C4—C3—H9	108.7 (16)	H17—C7—H18	107 (2)
H8—C3—H9	109 (3)	C8—C7—H16	107.5 (18)
C4—C3—H7	113 (2)	H17—C7—H16	111 (2)
H8—C3—H7	103 (3)	H18—C7—H16	115 (3)
H9—C3—H7	108 (3)	C5—C8—C6	109.5 (3)
C3—C4—C1	108.1 (3)	C5—C8—C7	108.8 (2)
C3—C4—C2	108.9 (3)	C6—C8—C7	107.3 (3)
C1—C4—C2	109.0 (3)	C5—C8—C2D	112.25 (19)
C3—C4—C1D	111.70 (19)	C6—C8—C2D	109.20 (19)
C1—C4—C1D	110.1 (2)	C7—C8—C2D	109.7 (2)
C2—C4—C1D	109.0 (2)	H1W—O1W—H2W	106 (3)
C2B—N2—H4N	110.4 (12)		
			
N1—C1B—C1A—C1'	55.6 (2)	N2—C2B—C2A—C2'	62.84 (18)
C1B1—C1B—C1A—C1'	172.92 (15)	C2B1—C2B—C2A—C2'	179.82 (14)
C1B2—C1B—C1A—C1'	−63.9 (2)	C2B2—C2B—C2A—C2'	−56.36 (19)
C1B—C1A—C1'—O1	174.79 (15)	C2B—C2A—C2'—O4	104.87 (18)
C1B—C1A—C1'—O2	−5.7 (2)	C2B—C2A—C2'—O3	−73.4 (2)
N1—C1B—C1B1—C1G1	−170.80 (15)	N2—C2B—C2B1—C2G1	−169.63 (16)
C1A—C1B—C1B1—C1G1	71.3 (2)	C2B2—C2B—C2B1—C2G1	−52.3 (2)
C1B2—C1B—C1B1—C1G1	−54.4 (2)	C2A—C2B—C2B1—C2G1	73.4 (2)
C1B—C1B1—C1G1—C1D	57.2 (2)	C2B—C2B1—C2G1—C2D	56.6 (2)
C1B1—C1G1—C1D—C1G2	−56.2 (2)	C2B1—C2G1—C2D—C2G2	−57.4 (2)
C1B1—C1G1—C1D—C4	175.04 (17)	C2B1—C2G1—C2D—C8	174.07 (17)
N1—C1B—C1B2—C1G2	170.54 (15)	C2G1—C2D—C2G2—C2B2	57.3 (2)
C1B1—C1B—C1B2—C1G2	54.5 (2)	C8—C2D—C2G2—C2B2	−174.78 (17)
C1A—C1B—C1B2—C1G2	−69.7 (2)	N2—C2B—C2B2—C2G2	169.76 (15)
C1G1—C1D—C1G2—C1B2	56.1 (2)	C2B1—C2B—C2B2—C2G2	53.0 (2)
C4—C1D—C1G2—C1B2	−175.00 (16)	C2A—C2B—C2B2—C2G2	−71.6 (2)
C1B—C1B2—C1G2—C1D	−57.0 (2)	C2D—C2G2—C2B2—C2B	−57.2 (2)
C1G1—C1D—C4—C3	68.5 (3)	C2G1—C2D—C8—C5	63.8 (3)
C1G2—C1D—C4—C3	−57.6 (3)	C2G2—C2D—C8—C5	−61.3 (3)
C1G1—C1D—C4—C1	−51.7 (3)	C2G1—C2D—C8—C6	−174.6 (3)
C1G2—C1D—C4—C1	−177.7 (3)	C2G2—C2D—C8—C6	60.3 (3)
C1G1—C1D—C4—C2	−171.2 (3)	C2G1—C2D—C8—C7	−57.3 (3)
C1G2—C1D—C4—C2	62.8 (3)	C2G2—C2D—C8—C7	177.6 (2)

### Hydrogen-bond geometry (Å, º)

**Table:**

*D*—H···*A*	*D*—H	H···*A*	*D*···*A*	*D*—H···*A*
N1—H1*N*···O2	1.00 (2)	2.12 (2)	2.792 (2)	123.5 (15)
N1—H1*N*···O1*W*^i^	1.00 (2)	2.11 (2)	2.919 (2)	137.8 (17)
O1*W*—H1*W*···O1	0.89 (3)	2.03 (3)	2.903 (2)	166 (3)
N1—H2*N*···O4^ii^	0.96 (2)	1.81 (2)	2.747 (2)	166.0 (17)
O1*W*—H2*W*···O3^iii^	0.90 (4)	2.04 (4)	2.929 (2)	169 (3)
N1—H3*N*···O3	0.97 (2)	1.86 (2)	2.7903 (19)	160.7 (17)
N2—H4*N*···O2^i^	1.00 (2)	1.73 (2)	2.729 (2)	170.5 (19)
N2—H5*N*···O1^iii^	0.99 (2)	1.818 (19)	2.779 (2)	163.1 (18)
N2—H6*N*···O3	0.93 (2)	2.10 (2)	2.836 (2)	135.4 (17)

Symmetry codes: (i) −*x*, −*y*, −*z*; (ii) −*x*, −*y*−1, −*z*; (iii) −*x*+1, −*y*, −*z*.
